# Simulation of operating room crisis management - hypotension training for pre‐clinical students

**DOI:** 10.1186/s12909-020-02477-8

**Published:** 2021-01-18

**Authors:** Peng Gao, Chenyu Wang, Shijia Liu, Kevin C. Tran, Qingping Wen

**Affiliations:** 1 Department of Anesthesiology, First Affiliated Hospital of Dalian Medical University, NO.222 Zhongshan Road, 116011 Dalian, China; 2 Department of Physiology and Cell Biology, The Ohio State College of Medicine, 43210 Columbus, Ohio USA; 3 Department of Anesthesiology, Dalian Medical University, 116044 Dalian, China

**Keywords:** Pre-clinical, Anesthesia undergraduate students, Simulation, Hypotension

## Abstract

**Background:**

Simulation training is an essential criterion for medical staff. The majority of residents are trained in operating room crisis management (ORCM), but only a few pre-clinical anesthesia undergraduate students are trained. Anesthesia methodology and technology were studied by the anesthesia undergraduate students in theory, but they were not able to practically resolve all clinical problems scientifically and reasonably. Consequently, there is a need to apply their competencies and bring together their technology knowledge practically. The crisis management of operating room emergencies was a method of choice applied and used over time. Here, we designed the scenarios for comprehensive crisis management to train anesthesia undergraduate students. We tried to establish or identify the problems which occurred during attempts to implement these scenarios.

**Methods:**

Anesthesia undergraduate students initially examined the basic theory, fundamental practice techniques, and case studies before the simulation training program. Subsequently, they participated in comprehensive ORCM training. Training outcomes were evaluated through different viewpoints: understanding the subject, crisis management, nontechnical skills, and a user experience evaluation.

**Results:**

Anesthesia undergraduate students performed significantly better with completion of ORCM, indicated by higher scores in all four tests (*P* < 0.001), as well as clinical crisis management (*P* = 0.0016) and nontechnical skills (*P* = 0.0002). Following the simulation, the students described the experience as helpful in “combining theoretical knowledge with clinical practice”, helpful with memorization, and in “promoting understanding of the subject,” while “learning clinical logic authentically” and “inspiring learning interests.”

**Conclusions:**

This research indicates that ORCM could be implemented as a useful learning tool for pre-clinical anesthesia undergraduate students. The ORCM could be an excellent training method to help improve students’ professional competence in crisis management and nontechnical skills, integrating the knowledge and technology of the field of anesthesiology.

## Background

Advanced life support simulation training is an important prerequisite for medical staff [1]. Intraoperative resuscitation and crisis management require the engagement of a multi-professional, pre-formed perioperative team [2]. Simulation training is an essential part of teaching proper procedures in modern medicine [3–5], with the advantage of improving clinical skills while reducing adversely significant clinical events [6]. Simulation training for operating room crisis management (ORCM) is commonly used to teach residents [7, 8]. This training has great potential to improve patient safety, especially by lowering or eliminating medication errors during anesthesia administration [9, 10]. Few recently reported research projects focus on pre-clinical anesthesia training programs catered to undergraduate students. We wish to evaluate the impacts of ORCM simulation on the outcomes of pre-clinical anesthesia undergraduate students. The purpose of our research to evaluate if ORCM simulation training could provide a useful training tool for educating pre-clinical anesthesia undergraduate students. This could further lead to investigating crisis management training effects in the real operating room.

After graduation, pre-clinical anesthesia students could choose to participate in a standardized training program for residents or a master’s degree program. Most students experience comprehensive simulated scenarios during their standardized resident training programs, including ORCM anesthesia resources [11, 12]. In our anesthesiology program, undergraduate students must first study anesthesia’s basic theory while training their anesthesia-related skills. Unfortunately, this program cannot cover and address every problem which may arise in clinical practice. There is a need to implement ORCM simulation training of ORCM before entering clinical practice as interns. Intraoperative hypotension is a well-known negative effect of anesthesia, leading to a poor prognosis from different complications, such as postoperative organ injury, stroke, or even death [13–15]. An anesthesiologist needs to be familiar with circulation management, avoiding extreme intraoperative hypotension. Therefore, we aim to prepare and simulate severe hypotension scenarios under anesthesia through ORCM simulation training. ORCM was one of several established inductive instructional methods [16]. We aim to improve clinical practice by integrating aspects of anesthesiology theory from isolated and one-sided knowledge to systematic and comprehensive knowledge. This study will identify current limitations while enhancing students’ learning awareness through ORCM simulation training.

## Methods

### Ethics approval and consent to participate

The study was conducted through SimMan. No patients were involved or harmed in this study. The study was approved by the Teaching Management Department of Dalian Medical University (the Teaching Guideline, 2015 version). The curriculum strictly followed the standard for Dalian Medical University. Formal consent was obtained from all study participants.

### Normal training phase

Before beginning ORCM simulation training, anesthesia undergraduate students first completed anesthesiology theory courses, learning the foundational practice techniques. They must also complete various case study courses, demonstrating situations such as pheochromocytoma, anaphylactic shock, obstructed airway, and body temperature management, in order to improve logical thinking practice in a clinical setting. After completion of these programs, students will become involved in ORCM simulation training.

### Training scenarios

A simulated operating room was prepared, and four hypotension scenarios were designed. Topics consisted of decreased heart rate due to gallbladder reflex (Scenario 1), reduction of vascular peripheral resistance from anaphylactic shock (Scenario 2), hypovolemia by massive blood loss (Scenario 3), and decreased cardiac contractility secondary to arrhythmia in local anesthetic poisoning (Scenario 4).

### Training participants

Training participants were selected based on minimal experience or exposure to clinical anesthesia. In the current study, 31 students were selected. Several participants were eligible for clinical internship programs. The training session was conducted at the end of the 7th semester in the ten-semester-long anesthesia undergraduate program. This educational program provides clinical intern stages during the 8th and 9th semesters.

### Participant demographics

Demographic data for all training program participants were collected, including gender, age, qualified skill examination, qualified theoretical assessment, their ability to solve case problems, previous experience with simulated scenarios, and clinical experiences.

### Training setting

All participants were not informed of the simulation’s contents before training. All students were administered a pre-test, which consisted of a short, 15-minute written examination related to scenarios’ topics. Example questions were “What is a choledochal reflex,” and how can one identify and manage such, which is related to Scenario 1. Scenario 2’s related questions were asked to describe and identify anaphylactic shock and how to manage such. Regarding Scenario 3, students were asked to describe factors that affect blood pressure and how to reverse hypovolemia. For Scenario 4, students had to describe and identify local anesthetic poisoning while discussing steps to manage such. After completing the pre-test, students were randomly divided into groups and assigned roles with visual labels. The first three teams were comprised of 2 anesthesiologists, 2 surgeons, and 1 nurse, with each role having its own supervisor for eight members. The fourth team had only 7 members, lacking a supervising nurse.

**Fig. 1:**
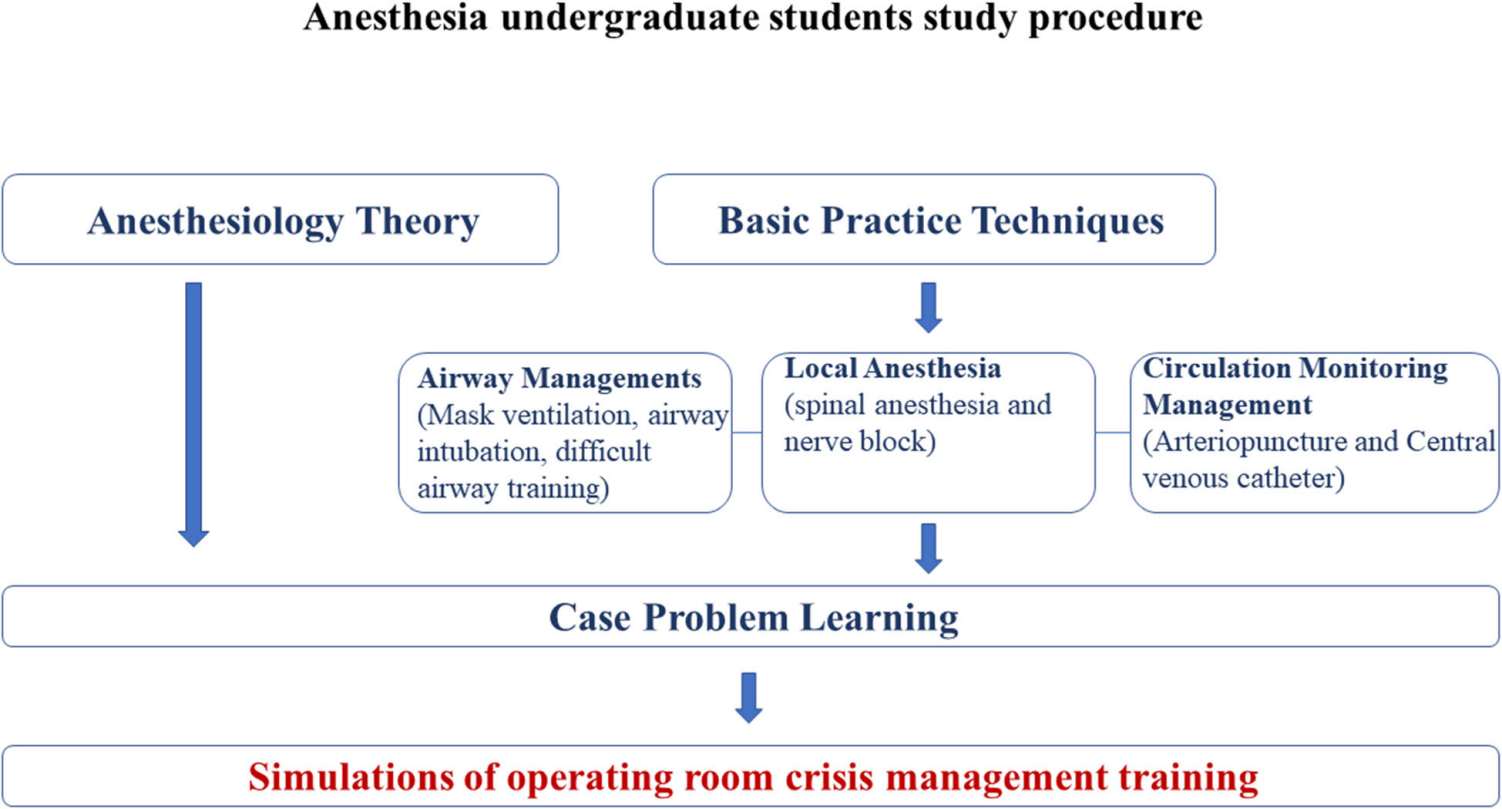
Anesthesia undergraduate students study procedure. The organizational chart for the procedure of the anesthesia students’ program is reflected in this Figure. The chart covers the basic practice techniques as well as the theoretical aspects of anesthesiology. All sections will be integrated into case study learnings before beginning the simulated training program

An operating room scene was prepared alongside the four scenarios of intraoperative hypotension. Each scenario lasted for 12 minutes, after which there was a 20-minute debriefing period. Students’ performances were analyzed and evaluated during this time. After completing the simulation training, a 15-minute final test and survey were administered (Fig. 1). Topics on the final test were identical to the ones found on the pre-test. Throughout the procedure, instructors did not provide examination answers, and students were unaware of a final exam. Examination results were kept hidden from participants, and all evaluations were administered in Chinese.

### Training format

Before the simulation, the instructors explained the whole process and how to proceed through simulation procedures to participating students. All four scenarios were administered to each of the four groups. Students performed treatments according to the simulated vital signs and the overall surgical operation described during the training process. Instructors were able to modify vital signs and operational progress within the simulation software. After completing each scenario, instructors and students summarized the progression of the scenario and discussed its results. If simulated patients suffered adverse effects, students were asked to identify if all necessary actions and precautionary measures were performed. Student performance was analyzed and evaluated during this time. Improvement plans were made during the discussion period. Simulation scenarios were repeated to gauge student progress and improvement as necessary.

### Evaluation

Students were evaluated and scored at the end of the scenario simulation activity. Evaluation of the whole process, especially crisis management points and nontechnical skills, was performed according to the evaluation standards found in Table 1. These standards are strictly based on the Emergency Manual by the Stanford Anesthesia Cognitive Aid Group [17] as a standardized procedure generally accepted by the anesthesiology community. The evaluation was open and transparent to all participants in the debriefing phase to facilitate effective analysis, evaluation, and comprehension of the discussions. Students were free to debate differing opinions until an agreement was reached. The crisis management was graded as the following: 2 points if the exercise was completed in its entirety, 1 point if more than 50% completed was completed, and half a point for less than 50% completion. Zero points were awarded for no work. Nontechnical skills included task management (such as assigning and ordering medical processes), teamwork (whether between anesthesiologists or anesthesiologists and other medical personnel), communication, sustained vigilance (such as anticipation and concentration), reaction time (time to respond to changes in condition), crisis identification (judgment of etiology), decision making (accuracy of the decision and promptly), and self-confidence (belief in oneself and calm judgments). One point was awarded for perfect completion or satisfaction in each category, half a point for partial success, or zero points if a student lacked in said area. Each category of nontechnical skills was analyzed in addition to an overall total score. All pre-tests and final tests were independently evaluated by three separate instructors.

**Table 1:** The simulation of hypotension of advanced life support in the operationroom

Classification	Scenarios	Crisis Management Points	Nontechnical skills	Score
**Hypotension**	Heart rate decrease (Vagus reflex)	Block of the afferent and efferent nerve (Lidocaine-*2 points*)	Task management Teamwork Communication Sustained vigilance Fast reaction time Crisis identification Decision making Self-confidence (*1 point each task*)	
		Neural receptor (Stop operation-*2 points*)		
		Neural effector (Atropine-*2 points*)		
	Reduction of vascular peripheral resistance (Anaphylactic shock)	Allergen (Stop injection-*2 points*)		
		Antiallergic (Drugs-*2 points*)		
		Hemodynamic maintenance (Vasoactive agent, blood volume-venous passage, infusion speed-*2 points*)		
	Hypovolemia (Massive blood loss)	Cause of disease (Quick judgment and processing-*2 points*)		
		Hemodynamic maintenance (Vasoactive agent, blood volume-venous passage, infusion speed-*2 points*)		
		Blood transfusion preparation (component blood transfusion, Autologous blood transfusion-*2 points*)		
	Cardiac contractility (Local anesthetic poisoning)	Cause of disease (Quick judgment and stop injection, benzodiazepine-*2 points*)		
		Basic life support (adrenaline, airway and breathing-*2 points*)		
		Injection of fat milk (if necessary-*2 points*)		
		Management of arrhythmia and hypopiesia-*2 points*		

Remarks: Crisis Management Points-score *2 points* for 100% completed, *1 point* for more than 50%, *0.5 point* for less than 50%, and *0 point* for zero completed

Nontechnical skills- score *1 point* for a perfect completed, score *0.5 point* for a partially completed, and *0 point* for zero completed

### Use of the equipment or props scene

The model used was from Jucheng (Yingkou, China), and monitors ran either SimMan or Vital Sign Simulator software. Scene props, such as an anesthesia machine and vitals monitors (ECG, sphygmomanometer, pulse oximeter) were present. Simulated therapeutic props, such as a laryngoscope, tracheal tube, central venous catheter package, artery puncture needle, ultrasound readers, syringe, and various imitative drugs (such as anesthetics, vasoactive drugs) were also used. During simulation re-runs or additional attempts, students were able to select the appropriate props to pursue relevant and necessary measures.

### Statistical analysis

All tests were blindly graded to facilitate evaluation strictly based on the established standards and previously mentioned scoring guidelines. The scores and time data were analyzed through the nonparametric Wilcoxon matched-pairs signed-rank test. The sample was based on a pair for a “before-and-after” study, 2-tailed and confidence intervals were 95%. A *P* value less than 0.05 was considered statistically significant.

These groups and scenarios were randomly assigned. The crisis management points and nontechnical skills were evaluated by each of the 4 scenarios. Scores and time data from subsequent scenario trials were paired for a “before-and-after” study (the time from the first attempt at a scenario compared with the second attempt). Data were analyzed with paired t-test. *P* < 0.05 was considered statistically significant. All the results were analyzed using the GraphPad Prism 5 software.

## Results

**Fig. 2:**
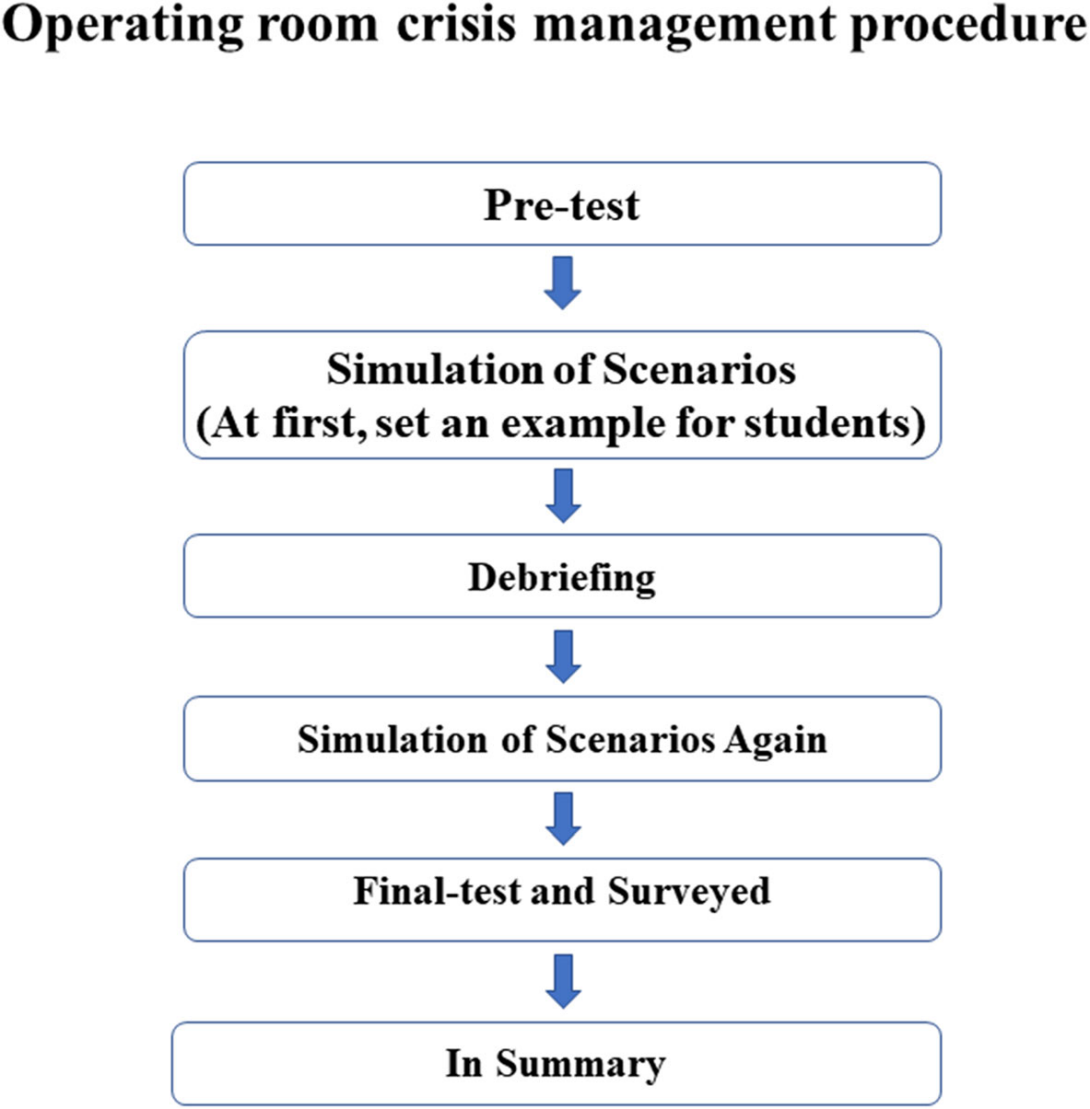
Operating room crisis management procedure. The chart about ORCM hypotension training procedures reflects all the steps expected to be covered and executed in this training program

The organizational chart for the anesthesia students’ program is reflected in Fig. 2. The chart covers the basic practice techniques as well as theoretical aspects of anesthesiology. All sections will be integrated into the case-study program as important steps before the simulated training program. The ORCM hypotension training procedure chart reflects all the steps expected to be followed in this training program (Fig. 1).

The simulated scenarios of hypotension under advanced life support within the operating room are presented in Table 1. Scenarios that can lead to hypotension are listed here: decreased heart rate, vascular peripheral resistance reduction, hypovolemia, and decreased cardiac contractility. These scenarios all have crisis management points that need to be addressed by students. Nontechnical skills and assigned scores are detailed for each scenario.

The demographic details for all participants are presented in detail in Table 2. It is important to note that all selected students had already passed theoretical and technical practice examinations and completed the case studies. However, these students had no clinical or simulated scenario experience. The group of students participated in a written test in order to be familiar with the content of intraoperative hypotension factors, heart rate decrease (gallbladder reflex and heart rate decrease), reduction of vascular peripheral resistance (anaphylactic shock), hypovolemia (massive blood loss), and cardiac contractility decrease (arrhythmia by local anesthetic poisoning). Of note, students’ scores significantly improved from their pre-tests to their final test. Hypotension pre-test and post-test scores are summarized in Table 3. The final test revealed that most students improved their responses and were able to detect and explain the key points regarding basic theory. This was reflected by significantly improved examination scores from the pre-test to the final test (Table 3). Additionally, students needed significantly less time to finish their final test (Table 3). Similar observations were noted for repeated scenario simulations, where the second simulation was completed significantly faster than the first time (Table 4). The non-technical skills objective information and additional details are shown in Table 5.

**Table 2:** Demographics

Variable	Number	Percentage (%)
**Gender**		
Female	21	67.7
Male	10	32.3
**Age**		
26	1	3.2
25	1	3.2
23	3	9.7
22	8	25.8
21	17	54.8
20	1	3.2
**Normal Training Phase**		
Theory qualified	31	100
Technical qualified	31	100
Finish CBL learning	31	100
**Scenario of simulation experiences**		
Yes	0	0
No	31	100
**Clinical experiences**		
Yes	0	0
No	31	100

**Table 3:** Evaluation of pre-test and post-test about hypotension

Objects	Results		
	**Pre-test**	**Post-test**	***P value***
Choledochal reflex of judgement and management	3.086 ± 2.664	9.581 ± 0.9228	*P* < 0.0001
Factors of affecting blood pressure	5.387 ± 2.044	8.968 ± 1.426	*P* < 0.0001
Anaphylactic shock of judgement and management	4.097 ± 2.534	8.355 ± 2.138	*P* < 0.0001
Local anesthetic poisoning and how to judgement and management	4.613 ± 2.044	9.677 ± 0.5408	*P* < 0.0001
Test Time	10.61 ± 2.349 (min)	8.348 ± 1.747 (min)	0.0014

**Table 4:** Evaluation in simulation progress

Objects	Results		
	**First time**	**Second time**	***P value***
Nontechnical skills Scores	3.25 ± 0.2887	6.5 ± 0.4082	*P* = 0.0002
Crisis management Points	3.375 ± 0.4787	6.0 ± 0.7071	*P* = 0.0016

**Table 5:** Nontechnical Skills Objects Information

Nontechnical skills objects	First time				Second time			
	Mean ± SD	Minimum	Maximum	Degree of Difficulty^**a**^	Mean ± SD	Minimum	Maximum	Degree of Difficulty^**a**^
Task management	0.375±0.25	0	0.5	0.625	0.875± 0.25	0.5	1	0.125
Teamwork	0.375±0.25	0	0.5	0.625	0.875±0.2887	0.5	1	0.125
Communication	0.375±0.25	0	0.5	0.625	0.75±0.2887	0.5	1	0.25
Sustained vigilance	0.5±0	0.5	0.5	0.5	0.875± 0.25	0.5	1	0.125
Fast reaction time	0.375±0.25	0	0.5	0.625	1± 0	1	1	0
Crisis identification	0.5±0	0.5	0.5	0.5	0.75±0.2887	0.5	1	0.25
Decision making	0.5±0	0.5	0.5	0.5	0.875± 0.25	0.5	1	0.125
Self-confidence	0.25±0.2887	0	0.5	0.75	0.625± 0.25	0.5	1	0.375

^a^Degree of Difficulty 1 point-average / total points

**Fig. 3:**
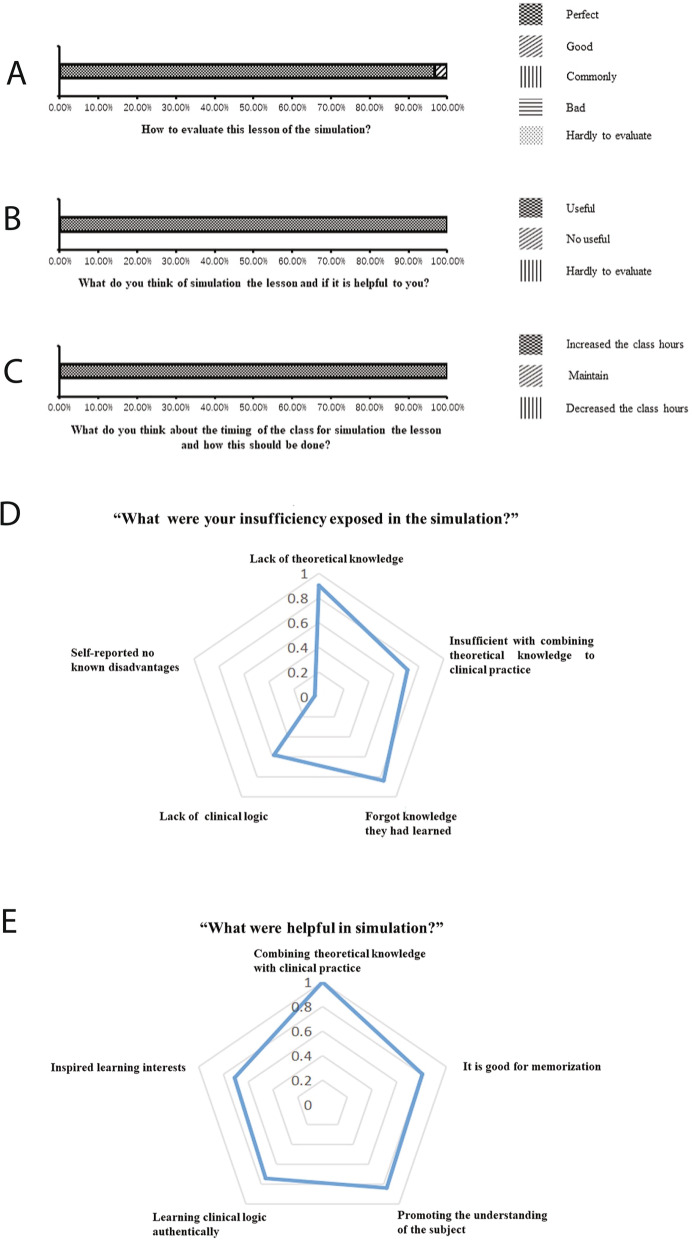
The simulations’ survey and evaluation of students’ responses. The questions implemented in this survey were listed as follows: “How would you evaluate this lesson of the simulation?” (**a**); “What do you think of the simulation lesson and was it helpful to you?” (**b**); “What do you think about the timing of the simulation lesson and how should this be done?” (**c**); “What were insufficiencies you experienced during the simulation?” (**d**); “What was helpful in the simulation?” (**e**)

The students’ comments on the learning experience obtained from the evaluation showed that the majority (96.8%) of the students selected described the experience as “perfect, improves the practice of theoretical anesthesiology as well as clinical outcomes.“ Only a few students (3.2%) chose rated the experience as “Good, helpful in improving their knowledge.“ Of note, no participants described the experience neutrally, unhelpful or unnecessary, or as a negative experience (Fig. 3A). Most, if not all, students believed that the simulation training program was “beneficial for studying” and “it is necessary to increase the time allocated for those classes” (Fig. 3b and c).

Regarding the multiple-choice question “What were your insufficiencies exposed in the simulation?” the majority of students (90.3%) chose “Lack of theoretical knowledge” while a different 71% of students chose “Insufficient with combining theoretical knowledge to clinical practice” 83.9% of students reported they “Forgot knowledge they had learned” and approximately half of the students (58.1%) chose “Lack of clinical logic” A small group of students (3.2%) self-reported no known disadvantages (Fig. 3d).

Another multiple-choice question was, “What was helpful in the simulation?” All students agreed that the simulation was most helpful in “Combining theoretical knowledge with clinical practice” A large percentage of students (80.6%) stated, “It is good for memorization” whereas 83.9% of students agreed that the simulation was helpful in “Promoting the understanding of the subject” A good percentage of students (74.2%) found the simulation helpful in “Learning clinical logic authentically,” and a similar percentage (71.0%) of students reported that the simulations “Inspired learning interests” (Fig. 3e). Overall, the ORCM hypotension simulation training program received good feedback with helpful highlights.

## Discussion

In recent studies, simulation medical training allows a learner to experience success, receive feedback, and gain confidence in a safe environment [18]. One of the studies indicated that in comparison to traditional learning techniques, simulation learning might have a greater effect [19]. Studies show how difficult the transition is from a medical student to a newly qualified doctor making decisions based on clinical logic [20]. Our teaching is based on how anesthesia undergraduate students study the basic theory, methodology, and skills. Their knowledge was isolated and scattered, especially because they lacked relevant logical thinking [21–23]. Undergraduate students had limited clinical experiences and were weak in clinical logic and diagnosing ailments. Undergraduate students do not have the flexibility of using professional anesthesia knowledge correctly, mainly due to a lack of knowledge in methodologies. Hypotension is a well-known common adverse effect during interoperation, and it can cause operating room emergencies and related complications [24, 25]. Therefore, we have designed ORCM hypotension scenarios to improve clinical logic and help students make correct clinical decisions in particular clinical conditions.

In our study, we found the majority of students already knew the basic theory about the induction of hypotension through decreased heart rate (vagus reflex), reduced peripheral vascular resistance (anaphylactic shock), hypovolemia (massive blood loss), or decreased cardiac contractility (arrhythmia by local anesthetic poisoning). Final test scores were significantly higher than pre-test scores. Remarkably, students used less time to finish the final test compared to the pre-test. This emphasizes the benefits of systematically learning important knowledge points through ORCM simulation teaching. Scenarios were administered multiple times, each time with some improvement. However, the improvements manifested differently, such as students agreeing on different treatment parameters according to their previous interests or observations. Such instances were repeated for further evaluation. Another important point raised by students was their belief that the simulation classes were a better experience than traditional classes. The teaching process is a two-way street, with students providing feedback to professors’ lectures or teachings. It is essential to promote educational progress by focusing on instruction while being receptive to students’ needs for effective progress in their studies. To implement and assess learning experiences and their effects, the evaluation is an essential and useful tool. In the evaluation used, all students reported that the simulation was “beneficial for studying” and “time allocated for those classes must be increased.” Together, these observations suggest that the ORCM simulation training program greatly impacts pre-clinical anesthesia undergraduate students. This simulation training program helps students in improving and integrating anesthesiology theory with clinical practice.

The simulation has been used extensively to effectively train skills, knowledge, and teamwork principles related to clinical issues [26]. Our research indicates that simulations also improve anesthesia undergraduate students’ management of technical and nontechnical skills. Meanwhile, another evaluation demonstrated that most students agreed that simulation training helped them to “combine theoretical knowledge with clinical practice.” The students’ knowledge went from scattered and one-sided to systematic and comprehensive through debriefing. The standard process was summarized in the main points (such as show they can do well or improve). Therefore, ORCM simulation is a great training tool in linking theoretical knowledge with clinical practice. However, the students’ shortages were also reported in our simulation process, such as a “lack of theoretical knowledge”, “insufficient in combining theoretical knowledge with clinical practice,” or “lack of clinical logic.” A high percentage of the students (71%) believed that this simulation training program would inspire their learning awareness.

Furthermore, the shortage of teaching programs was revealed through the simulation process. In operating room crises, good patient survival rates and prognoses required the anesthesiologists to possess advanced nontechnical skills competencies [27, 28]. The simulation tool could effectively create confidence, communication, and leadership skills for the students who attended [5, 29, 30]. In this study, the score obtained on nontechnical skills evaluation was significantly lower on the first attempt, possibly influenced by the universities’ educational methods. The methods primarily used are basic lectures and practice techniques, with few opportunities to train non-technical skills (such as situational awareness, decision-making, communication, teamwork, or leadership) [31]. Simulation training programs’ benefits can manifest as improvements in self-confidence, anxiety reduction, and greater belief in proficiency [32]. Therefore, non-technical skills are essential, but unfortunately, they are often neglected. Students may still miss some key points despite achieving better scores during the second evaluation of non-technical skills. A helpful course of action could be to expand instruction on non-technical skills and implementing these topics into daily learning.

This study has its own limitations. The research participant groups are only *n* = 31, and only four scenarios are used, which can contribute to bias in measuring all simulation training outcomes. The four scenarios are independently sampled for statistical analysis, which conflicts with the nontechnical details. Crisis management and nontechnical skills cannot be evaluated independently, which was the actual teaching condition. Nevertheless, we acknowledge that there is no control group and realize that it is more difficult to measure the simulation training outcomes. Our study requests that all students embrace the same teaching methods to guarantee fairness in grade evaluations. Our study was designed using a nonparametric self-paired test, and this reinforces our observations of significant self-improvement. We agree that surveys are not a comprehensive and objective evaluation indicator. Overall, given that this is one of the first studies on this topic, the authors believe that these results will begin to shed light on the benefits of ORCM simulation training for anesthesia undergraduate students.

Future research will have to examine the effects of scenario simulation training on undergraduate students. We plan to follow up on these students’ progress and growth through their medical careers. We also want to compare the differences between those who had clinical experience before the simulation and those who did not. To investigate the influence of simulation teaching, these observations would be performed at an earlier point in their careers.

## Conclusions

In summary, the simulation training program of ORCM can be an attractive and effective training methodology and a good tool for the pre-clinical anesthesia undergraduate students. It can also improve students’ abilities in combining knowledge of theoretical anesthesiology with clinical practice.

## Data Availability

The datasets used and/or analyzed during the current study are available from the corresponding author on reasonable request.

## Abbreviation

ORCM: Operating room crisis management
